# Sperm DNA Integrity and Male Fertility in Farm Animals: A Review

**DOI:** 10.3389/fvets.2020.00321

**Published:** 2020-06-19

**Authors:** Arumugam Kumaresan, Mohua Das Gupta, Tirtha Kumar Datta, Jane M. Morrell

**Affiliations:** ^1^Theriogenology Laboratory, Southern Regional Station of National Dairy Research Institute (ICAR), Bengaluru, India; ^2^Animal Genomics Laboratory, National Dairy Research Institute (ICAR), Karnal, India; ^3^Department of Clinical Sciences, Swedish University of Agricultural Sciences, Uppsala, Sweden

**Keywords:** male infertility, sperm, DNA integrity, epigenetic modifications, prognostic value

## Abstract

The accurate prediction of male fertility is of major economic importance in the animal breeding industry. However, the results of conventional semen analysis do not always correlate with field fertility outcomes. There is evidence to indicate that mammalian fertilization and subsequent embryo development depend, in part, on the inherent integrity of the sperm DNA. Understanding the complex packaging of mammalian sperm chromatin and assessment of DNA integrity could potentially provide a benchmark in clinical infertility. In the era of assisted reproduction, especially when *in-vitro* fertilization or gamete intrafallopian transfer or intracytoplasmic sperm injection is used, assessment of sperm DNA integrity is important because spermatozoa are not subjected to the selection process occurring naturally in the female reproductive tract. Although sperm DNA integrity testing measures a significant biological parameter, its precise role in the infertility evaluation in farm animals remains unclear. In this review, the earlier findings on sperm DNA integrity in relation to male fertility are compiled and analyzed. Furthermore, the causes and consequences of sperm DNA damage are described, together with a review of advances in methods for detection of sperm DNA damage, and the prognostic value of sperm DNA quality on male fertility.

## Introduction

Infertility has been a concern throughout the ages and is still a significant problem in several species, including human beings, and farm animals. In spite of several advancements in diagnostic techniques and infertility treatments, the levels of infertility in the human population were similar in 1990 and 2010, with only a slight overall decrease in primary infertility (0.1%) and a modest overall increase in secondary infertility (0.4%) (1). Although such large-scale studies are very limited in farm animals, existing information indicates that subfertility is rising in livestock, which can have adverse effects on animal welfare and farm economy by delayed calving intervals and increased culling of animals. Dobson et al. (2) reported that, over the past 30 to 50 years, the first-service-pregnancy-rate in dairy cattle dropped from 70 to 40%. Intrinsically, both male and female contribute to infertility, but male factor infertility accounts for 40–50% of infertility (3). In crossbred bulls, it was reported that the “acceptable quality semen producing ability” decreased, over a period, from grandsire through sire to male progeny (4). The significance of male fertility is highly amplified in farm animals since semen from a male is used for artificial insemination in several thousand females, and use of semen from infertile bulls affects conception, leading to considerable losses for farmers.

Male infertility refers to a male's inability to achieve pregnancy in a fertile female. In human beings, it was observed that male factor alone is responsible for ~26% of infertility cases (5) and contributory in another 30–40% of infertile couples (6). Several causes have been attributed to male infertility including genetic disorders, hormonal disturbances, disorders in spermatogenesis, poor sperm quality and sperm DNA fragmentation (6, 7). In the last few decades, there has been an increasing body of research investigating the role of sperm DNA integrity in male factor infertility (8). Evidence is accumulating that DNA damage is greater in spermatozoa of infertile males compared to fertile males (9–12). In dairy bulls, it was reported that DNA damage accounted for significant variations in fertility and the proportion of spermatozoa with DNA damage was more than 2-fold higher in below-average fertility bulls compared to above-average fertility bulls (13). Very recently, Boe-Hansen et al. (14) also confirmed the role of sperm DNA integrity in bull fertility and suggested that the presence of immature spermatogenic cells, cytoplasmic proximal droplets and alterations in sperm head shape were associated with sperm DNA integrity and protamine deficiency. Similar reports on the role of sperm DNA integrity in fertility and/or semen quality are available for other farm animals, including stallion (15, 16), boar (17–19), and ram (20, 21). Interestingly, sperm DNA integrity in relation to male fertility has become a hot topic recently. A search using the keywords “sperm DNA integrity and fertility” in PubMed showed 5,700 publications on this subject between 2005 and 2019, in contrast to only 251 publications between 1990 and 2004. On the other hand, despite the higher number of publications, there is no consensus on whether measurement of sperm DNA damage provides any clinical benefit in the assessment of male infertility (22).

In this review, previous findings on sperm DNA integrity in relation to male fertility are compiled and analyzed. Furthermore, the causes and consequences of sperm DNA damage are described, together with a review of advances in methods for detection of sperm DNA damage, and the prognostic value of sperm DNA quality on male fertility is also discussed.

## Sperm DNA Integrity and Causes of Damage

Since the findings of Alfert (23), that histone is replaced by protamine during spermiogenesis in salmon, research on sperm DNA integrity and fragmentation in relation to fertility increased rapidly. The pioneering work of Evenson et al. (24) on the relationship of DNA integrity and pregnancy outcome showed the existence of significant variations in sperm DNA integrity among individual males. Intact DNA is defined as the complete absence of nicks or breaks, either single or double-stranded, or any chemical modifications in its structure (25). Generally, most of the affected spermatozoa are phagocytosed by Sertoli cells or undergo programmed cell death by a caspase-dependent apoptosis pathway without releasing any harmful substances (26, 27). Despite this safety mechanism, sperm DNA damage occurs, which might be due to the liberation of substances such as reactive oxygen species (ROS), amongst others, from the dead spermatozoa. However, the findings of several researchers confirmed two possible causes of damage: one during spermatogenesis, due to impaired chromatin maturation, and the other during spermiogenesis, at the time of histone replacement by protamine followed by further compaction of DNA during epididymal transits (28, 29). Most of the damage is premutagenic and could be a result of either intrinsic or extrinsic factors.

## Intrinsic Factors

### Insufficiency in Recombination During Spermatogenesis

Usually, in a homologous pair of chromosomes, crossing over of genetic material during spermatogenesis takes place by activation of specific nucleases, which favors DNA breaks/damage. Thus, any kind of aberration in the recombination process can lead to cell death e.g., DNA–DNA and DNA-Protein cross-linking occur in the highly compact chromatin of mammalian germ cell rather than in somatic cells and is more commonly seen in defective spermatozoa (30).

### Reactive Oxygen Species

Reactive oxygen species (ROS) are highly reactive and retain the capacity to damage any cell structure or function. They are produced in both fertile and infertile individuals and have positive as well as negative effects. Their production in semen is controlled by both the spermatozoa and the seminal plasma antioxidant defense system. Spermatozoa possess low intra-cellular antioxidant activity consisting of superoxide dismutase, glutathione peroxidase, peroxiredoxin, thioredoxin, thioredoxin reductase etc., depending on the species. In stallions, spermatozoa also have intrinsic antioxidant defenses, such as glutathione and various enzymes such as paraoxonase, thioredoxin and the peroxidation families of proteins (31). Pathogenic effects are observed when the production of ROS exceeds the capacity of the antioxidant defense system to neutralize them or there is infiltration of leukocytes (32). Excessive levels of ROS coupled with a deficiency in antioxidants can lead to oxidative stress resulting in nuclear and mitochondrial DNA damage, telomere shortening, epigenetic alterations and Y chromosomal microdeletions (33). Bovine sperm DNA integrity is affected by excessive exposure to ROS, obliterating DNA compaction or the repair of double- or single-stranded breaks during the reconfiguration of DNA, as occurs in cryopreservation of semen samples (34, 35). A single-strand break is the outcome of an oxidative attack, whereas a double-strand break is the indirect consequence of the products of lipid peroxidation e.g., 4- hydroxyl-2-nonenal (36).

### Abortive Apoptosis

DNA damage due to apoptosis mainly occurs in the testis during spermatogenesis. Apoptosis controls the overproduction of spermatozoa and restricts the proliferation level during unfavorable conditions for sperm development. The presence of apoptotic markers in mature sperm, including Fas, Bcl-X, p53, and annexin V all support the role of apoptosis in the generation of DNA breaks (37). However, the relationship between the presence of these typical markers of apoptosis in spermatozoa and the degree of DNA fragmentation is not straightforward. It is reported that the routes for sperm DNA fragmentation and cell death are not fully caspase dependent (38). Abortive apoptosis is a phenomenon where the defective sperm cell escapes programmed cell death and is present in the ejaculate. However, these defective sperm cells with partial DNA breaks retain their fertilizing potential but lack the ability to support pregnancy, resulting in early embryonic loss (39, 40).

### Endogenous Nuclease

During spermatogenesis, endogenous nuclease (topoisomerase II) is activated, relieving torsional stress of highly compacted chromatin packaging in immature spermatids. The replacement of 85–95% of the histones by transitional proteins and eventually by Protamine 1 and 2 occurs, resulting in further condensation of chromatin materials and cessation of transcription and translation (41). However, during this replacement process, endogenous nucleases (topoisomerase II) create and ligate nicks, with the result that the majority of the damage is repaired during epididymal transit. Sometimes nicks in sperm chromatin escape this repair mechanism, with the result that spermatozoa with damaged chromatin appear in the ejaculate, indicating aberrant spermatogenesis and incomplete maturation. Normally, the ratio of protamine 1 to total protamine in spermatozoa varies widely among species; 0 for bull, 0.14 for stallion, 0.34 for hamster, 0.43 for human, and 0.67 for mouse (42). On the other hand, the relative proportion of sperm protamine 1 to sperm protamine 2 is nearly similar, with a ratio of 1:1 in human, bull, stallion, boar, and rodents. However, any alteration in this ratio is associated with DNA fragmentation, poor fertilization and reduced conception rate (43).

## Extrinsic Factors

### Age

While a few studies suggest that sperm DNA integrity is not affected by age (44, 45), other studies demonstrate a significant increase in sperm DNA damage with age (46, 47). In contrast, Fortes et al. (48) found that younger bulls had higher DNA fragmentation indices compared to adult animals. In Nellore bulls, it was reported that young bulls (1.8–2 years) and aged bulls (8–14.3 years) were found to be more susceptible to DNA damage compared to adult bulls (3.5–7 years), with young bulls exhibiting more defective protamination than older animals and aged bulls showing more nuclear oxidative damage (49).

### Increased Testicular Temperature

Sperm DNA fragmentation was also reported to increase with increase in testicular temperature. Basically, maintaining scrotal surface temperature at 2–6°C lower than core body temperature is a prerequisite for normal spermatogenesis. Elevation of testicular temperature increased testicular metabolism and altered the hormonal profile, with concomitant rise in oxygen demand to sustain aerobic metabolism, resulting in tissue hypoxia, generation of reactive oxygen species, and deterioration of semen quality (50). Production of excessive ROS causes major damage to sperm DNA integrity (51). Several researchers observed a decrease in sperm count, sperm motility, normal morphology and increase in sperm DNA damage due to increase in thermal stress and environmental pollution in bull, horse, goat, human, mouse (52, 53).

### Cryopreservation and Storage Temperature of Sperm Samples

The effect of storage temperature and cryopreservation on sperm DNA have been studied in various species. Basically, temperature shock during cryopreservation are associated with a subsequent increase in oxidative damage to sperm DNA, plasma membranes and a decrease in viability, simultaneously leading to a decline in sperm quality. There is a dramatic increase in DNA fragmentation due to increased production of ROS during cryopreservation and storage of sperm cells for short or long periods of time in bull (54), ram (55), stallion (15), and boar (56). Various studies on sperm DNA fragmentation dynamics before and after sperm storage showed that cryopreservation is an important issue that must be considered since it can decrease DNA longevity (57, 58).

### Vaccination Stress

The effect of vaccination on semen quality has been widely studied. In general, vaccination stress causes febrile condition and elevates testicular temperature with a subsequent rise in ROS production. This increase in ROS, causes a deterioration in sperm morphology, antioxidative profiles and sperm DNA (59). In farm animals, it has been shown that vaccination-induced thermal insult resulted in extensive damage to the sperm DNA, reducing the fertilization rate (59, 60). Gosálvez et al. (61) also recorded a significant increase in sperm DNA damage after vaccination of rams, but observed that the negative impact was reversible. In another study, Gupta et al. (62) reported a deleterious effect of foot-and-mouth disease vaccine on plasma membrane integrity and morphology of bull sperm.

### Sex Sorting of Spermatozoa

During the recent past, the use of sexed semen for skewing sex ratio increased dramatically, especially in cattle. Some studies indicated that sex sorting does not affect the quality of sperm DNA in species such as deer (63) and boar (64). However, another study reported that although DNA fragmentation immediately after thawing was higher in conventional than in sex-sorted sperm samples, a reduced DNA longevity in sex-sorted spermatozoa was detected when the samples were incubated for 48 h (65). Using the Sperm Chromatin Structure Assay (SCSA) assay, it was shown that sex-sorted spermatozoa had less homogenous sperm chromatin than controls (66). Flow cytometric sexing has also been shown to induce high levels of ROS in sperm samples. In stallion spermatozoa, the DNA fragmentation index post-sorting were ~10% higher than pre-sorting, which may be due to oxidative DNA damage (67). Factors such as high pressure and speed, during sorting, dye-induced defects, electrical deviation, changes in pH and osmolarity, etc might lead to changes to the sperm plasma membrane, trigger pre capacitation-like changes, cause protamine decondensation and alter sperm DNA quality (68).

### Method and Season of Semen Collection

The method of semen collection has an effect on sperm DNA quality; in farm animals, it was shown that use of an artificial vagina method was superior, in terms of sperm DNA integrity, to electroejaculation for semen collection. Similarly, seasonal variations, semen dilution and washing protocols to harvest good quality sperm cells also had a detrimental effect on the integrity of sperm DNA (69, 70). Further, comparatively lower DFI% was observed in semen ejaculates collected during the breeding season as compared to the ejaculates obtained during the non-breeding season in bucks.

### Infection of Male Reproductive Organs

Acute or chronic inflammation of male accessory sex glands is associated with a significant increase in ROS causing a marked reduction in semen quality and apoptosis of sperm cells (71). In human beings, it was shown that bacterial infections had significant negative effects on sperm chromatin condensation and protamine P1/P2 ratio (72). In bulls, sperm DNA fragmentation increased because of bacterial growth during incubation of frozen-thawed semen. However, the increase in sperm DNA fragmentation was characteristic of some bulls but was not observed for others (73), indicating individual variations in susceptibility.

## Why Test Sperm DNA Damage—Consequences of Sperm DNA Fragmentation on Fertility

**Figure 1:**
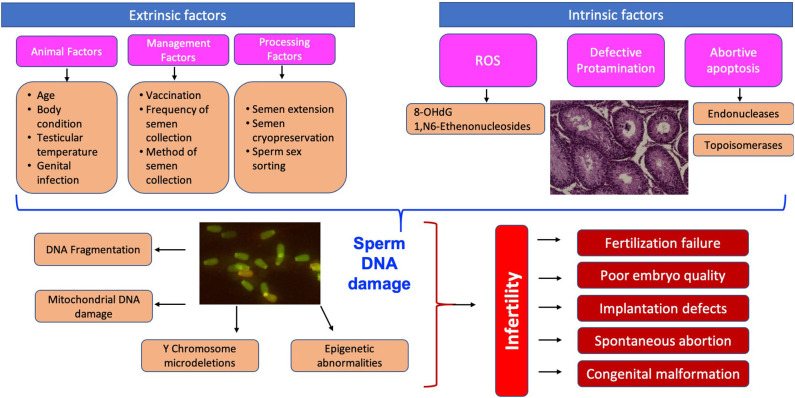
Schematic representation of the causes and consequences of sperm DNA damage.

Sperm DNA is the only male heritable material present at the time of fertilization; therefore, transfer of sperm with damaged DNA can result in deleterious effects on the conceptus and impact on the successful development of an offspring too (74). The schematic representation of the causes and consequences of sperm DNA damage is given in Figure 1. The presence of sperm DNA as highly condensed chromatin renders it transcriptionally and translationally silent besides protecting the DNA from damage (75). Despite its highly complex structure, sperm DNA is susceptible to damage and nearly 80% of infertility cases due to idiopathic reasons are related to DNA integrity (11). The proportion of spermatozoa with DNA fragmentation was 34.5% in infertile men while it was only 14.9% in fertile men (76). Assessment of sperm DNA fragmentation represents a promising tool for clinical and research practice (77), although larger prospective trials are needed. Hitherto, it was believed that due to the high compaction of chromatin material in spermatozoa there is limited scope for DNA repair.

The DNA fragmentation index (DFI) is defined as the percentage of spermatozoa with either abnormal protamination or with DNA damage (78). Alterations in chromatin packaging/DNA integrity can occur at any stage, starting from the germ cell during spermatogenesis to ejaculated spermatozoa (28). Surprisingly, the oocyte retains the capacity to repair such damage, depending on type and degree, suggesting that spermatozoa with abnormal DNA retain the capacity to fertilize an oocyte. However, in most cases, such repair is followed by early embryonic mortality, implantation defect, chromosomal aberration and comparatively higher abortion rate (27). Furthermore, damaged DNA is negatively correlated to fertilization, implantation, pregnancy outcome and the well-being of the male reproductive tract (79, 80). Variations in DNA integrity and fertility level among individuals with a normal spermiogram are also quite high in mouse (81), men (82), bulls (83), stallions (19), and boars (17). In bulls, it was reported that the proportion of morphological normal spermatozoa was negatively correlated to DFI. Sperm DNA integrity in bulls show associations with morphological parameters, particularly with head shape abnormalities and indicators of spermatogenic immaturity, including proximal droplets (14). Fortes et al. (84) reported that sperm protamine content and sperm DNA damage are closely associated. In contrast, Castro et al. (83) indicated that protamine deficiency in bovine spermatozoa may not have a strong biological impact in explaining difference of *in vitro* fertility between bulls. Kipper et al. (85), evaluated sperm chromatin packing in relation to *in vitro* fertilization success rate in Nellore bulls and found that the proportion of spermatozoa with abnormal chromatin compaction did not interfere with early embryonic development. In boars, sperm DFI had a significant negative correlation with farrowing rate and the average number of pigs born per litter (86). However, more detailed information is required to better understand the relationship between DFI and boar fertility.

Eventually, with the increase in male infertility and pregnancy failure, Evenson et al. (24) used sperm chromatin structure assay (SCSA) for detection of qualitative DNA damage as an indicator of male fertility. In human beings, a DNA fragmentation index (DFI) ≥ 20 % is associated with low fertility. Inseminating spermatozoa with compromised DNA, or performing assisted reproductive techniques (ART) without knowing the DNA status, can have a deleterious effect on fertility (9). Since then, a plethora of studies (11, 28, 87) indicated the existence of a substantial correlation between DNA damage and idiopathic (in)fertility. These studies concluded that spermatozoa in the neat ejaculate with sperm DNA fragmentation (SDF) values below 15% should be regarded as normal for this parameter, those ranging from >15% to <30% are likely to have some fertility problems, whereas individuals with levels of SDF >30% are considered to have substantial problems in producing offspring through natural conception (37). Sperm DNA assays could be one of the promising tools in selecting bulls for high fertility, since preliminary reports on sperm DFI and bull fertility indicate a significant relationship between these two parameters (13). Very recently, it was shown that measurement of DFI provides a simple, informative and reliable measure of sperm quality and can accurately predict male mouse fertility (81). These researchers observed that sperm DFI was significantly higher from males with low sperm counts compared to males with normal sperm counts, and viable embryos derived using spermatozoa from males with high DFI failed to produce offspring after embryo transfer compared to embryos from males with low DFI. Accumulated data show that DNA damage in mammalian species has biological consequences that vary according to the type of damage, location, cell or tissue involved. Such damage may induce temporary or permanent changes, either long-term or short-term. To improve the conception rate in artificial breeding and to reduce infertility in farm animals, it is essential to understand sperm DNA at the molecular level and the impact of sperm DNA integrity on field fertility.

## Sperm Chromatin Structure During and After Spermatogenesis

Spermatogenesis is a complex process involving several mitotic and meiotic divisions, followed by differentiation to produce fully mature haploid and polarized spermatozoa from a diploid spermatogonial stem cell. Spermatogenesis involves two steps, viz. spermatocytogenesis and spermiogenesis. Spermatocytogenesis is the process in which spermatogonia undergo proliferation, reduction division and differentiation to produce primary spermatocytes, secondary spermatocytes and finally spermatids. Subsequently, spermiogenesis involves a series of morphological transformations of immature spermatid to a mature spermatozoon. During the latter part of spermatogenesis, the chromatin material undergoes dramatic compaction, which renders spermatozoa transcriptionally silent, and cytoplasm is shed in the form of residual bodies (88). Meanwhile, the histone-DNA complexes of round spermatids are replaced by transition protein and then finally by protamine in elongated spermatids. Only 5–15% of histones are retained in DNA in spermatozoa from fertile men, whereas a comparatively higher proportion is retained in spermatozoa from infertile men (89). Nevertheless, these alterations instigate final condensation and stabilization of chromatin material, precisely portrayed in the mature phase of sperm cells. Sperm chromatin typically appears in a coiled fashion, called toroids, via formation of disulfide bonds (90) to protect DNA prior to fertilization. Around 50 kb of DNA is packaged into coiled toroids, attached to the nuclear matrix region by toroid linkers. Linker regions and histone-bound DNA are highly susceptible to DNA damage induced by endonucleases (91). Several researchers have demonstrated the negative effect of abnormal protamination in semen quality on the success of *in vitro* fertilization (IVF) and pregnancy rates in human patients (43, 92) and semen quality and fertility in bulls (93, 94). Furthermore, studies of Arpanahi et al. (95) and Hammound et al. (96) highlighted the significant role of retained histones in early embryonic development, zygotic genome activation, signaling pathways and imprinting genes, in the case of human and rat spermatozoa. In addition, it is documented that loss of chromatin integrity is associated with sperm morphological abnormalities (97), loss of viability and progressive motility (98), reduced concentration (45) and sperm maturity (99). Furthermore, Carrel and Liu (100) and Virro et al. (99) depicted a strong association between loss of chromatin integrity and poor implantation or spontaneous abortion. Therefore, the highly specialized sperm cell acts not only as a genetic carrier to the oocyte but also undergoes changes during spermatogenesis that will later support the developing embryo and maintenance of pregnancy.

## Epigenetic Modifications and Male Fertility

In the science of genetics, epigenetics is the study of changes in gene expression which occur without alterations in the DNA sequence. Basically, epigenetic changes involve interactions between DNA and protein, either short-term or inherited over a generation at various developmental stages of life. Spermatozoa have unique epigenetic modifications pertaining to DNA methylation, covalent histone modification, and chromatin remodeling (101). Such changes may be associated with either expression or silencing of genes endowed with beneficial or detrimental effects in an organism. Of all these epigenetic modifications, abnormal DNA methylation has emerged as one of the promising indicators of male infertility (102).

### DNA Methylation

During condensation of nuclear material in elongated spermatids, DNA methylation occurs in the cytosine residues of CpG islands under the influence of DNA methyl transferase enzyme. Methylation regulates genes expression either by adding (hypermethylation), or removing methyl groups from the promoter region (hypomethylation). Hypermethylation is attributed to the shutdown of gene expression or transcriptional silencing whereby the formation of protein comes to a halt and the developing zygote utilizes stored maternal mRNAs until the activation of the zygotic gene (103). Besides, several processes such as genomic imprinting (104), inactivation of the X chromosome (105), DNA compaction (106), and gene silencing are critically involved in DNA methylation of germ cells. However, during development and maturation of germ cells, extensive removal of DNA methylation in the primordial germ cell transpires (107). Prior to meiosis, compaction of chromatin in the sperm cell renders it much denser than in somatic cells (106). Any exposure to a deleterious environment may change DNA methylation patterns in male germ cells and interfere with differentiation into functional sperm cells, ultimately impairing male fertility. Any deviation in the methylation pattern limits the chances of successful fertilization and embryonic sustainability (108). A plethora of studies [e.g., (96, 109, 110)] have also confirmed a positive correlation between abnormal methylation and male infertility, having a considerable influence on various seminal traits and pregnancy outcome. Several studies indicate the relationship of methylation status of genes with fertility (Table 1). Jena et al. (111) reported the effect of abnormal methylation pattern on the expression of IGF2 and H19 genes, which result in disruption of spermatogenesis, apart from producing spermatozoa with altered epigenetic marks, poor sperm quality and compromised fertility in crossbred bulls. Recently, Kropp et al. (118) also reported that bull fertility status is associated with DNA methylation signatures in spermatozoa. These researchers observed that preimplantation embryos derived from high and low fertility bulls displayed significant transcriptomic differences and there were differences in methylated regions which could influence the reprogramming of the early embryo. Tunc and Tremellen (119) also detailed the significant variation in spermatogenic efficiency and quality of semen among fertile and infertile groups of individuals in relation to global DNA methylation. Furthermore, infertile individuals with aberrant DNA methylation-imprinting were reported to have marked oligoasthenoteratozoospermia and oligozoospermia (120).

**Table 1:** Methylation status of selected genes in relation to fertility.

**Genes**	**Aberration**	**Male infertility**	**References**
H19, IGF2	Hypomethylation	Oligozoospermia and infertility in crossbred bulls	(109, 111)
MTHFR	Hypermethylation	Poor semen quality and infertility	(112, 113)
PAX8, NTF3, SFN, HRAS	Hypermethylation	Oligozoospermia, teratozoospermia and asthenozoospermia	(114, 115)
RASGRF1	Hypermethylation at an imprinted locus	Poor semen parameters in boar, human and mice	(93, 116)
GTL2	Hypermethylation at an imprinted locus	Poor semen parameters	(115, 117)
PLAG1, D1RAS3, MEST	Hypermethylation at imprinted locus	Poor semen parameters in human and boar	(109, 115)
KCNQ1, LIT1, SNRPN	Hypermethylation at imprinted locus	Poor semen parameters in human and boar	(96, 115)

Techniques such as sex-sorting, vitrification and cryopreservation are reported to induce various degrees of DNA damage in bull spermatozoa (121). These processes result in abnormal methylation imprints which directly or indirectly reflect chromatin packaging (122) and, in turn, influence early embryonic developmental stages along with spermatogenesis of an embryo (123). When the status of DNA methylation was studied in boar sperm cells with different levels of DNA fragmentation, the number of differentially methylated cytosines was increased in the low-high compared to the low-medium and the medium-high DFI groups (19). These latter researchers concluded that with increasing DNA fragmentation in spermatozoa, there is an increase in the number of potentially affected downstream genes and their respective regulatory pathways. Although several researchers (109, 124, 125) have documented sperm and testicular methylation profiles in groups of fertile and infertile individual pertaining to promoter region, genes and imprinted regions, the mechanism elucidating such alteration and contributing to compromised fertility is still a matter of debate.

### Sperm Histone Modification

In mature spermatozoa, depending upon the species, between 2 and 15% of sperm chromatin is bound to histones, rather than to protamines (126). Although the ratio of nucleohistone to nucleoprotamine ratio is very low, it still plays a significant role in gametogenesis. Generally, histones are octameric and have four types of histone proteins in the core of the nucleosomes: H2A, H2B, H3, and H4. On the other hand, arginine- and lysine-enriched histone tails remain on the surface of the nucleosomes. Basically, all these histone proteins are susceptible to covalent modifications such as acetylation, methylation, phosphorylation, and ubiquitination. Most of these modifications occur under the influence of specific enzymes limited to the tail regions only, but modifications of histones in the core region are still under debate. However, recent research has elucidated the role of core histone modifications in the transcription process, as well as in the DNA repair mechanism, and replication and adjustment of cells. In addition, all the above post-translational modifications alter the DNA binding pattern, along with the interaction of other regulatory factors, and change the expression of genes (127). In other words, post-translational modification of histones is regulated by a number of molecules, which in turn modulate the chromatin architecture and may be involved in an alteration in fertility (Table 2). Gene activation or repression, instigated by methylation of histones, occurs under the influence of HMTase on H3 or H4. Moreover, Okada et al. (130) reported that methylation is an integral part of spermatogenesis. The progression of spermatogenesis is determined by the number of methyl groups added to H3K4, H3K9, or H3K27 (141), where methylation of H3K4 correlates with the functional competence of the spermatogonial stem cell to develop into fully mature spermatozoa, whereas methylation of H3K9 or H3k27 highlights gene silencing in the germ cell, eventually occurring after the commencement of meiosis. In light of this fact, any disruption to H3K9, demethylase JHDM2A (JmjC domain-containing histone demethylase 2A) and curtailment of the expression of protamine 1 and transition protein and decondensation of chromatin, subsequently result in infertility (130). Similarly, histone acetylation in elongating spermatids also plays a crucial role in the condensation of chromatin materials by replacing histone and transition protein by protamine; low hyperacetylation is reported in infertile men with aberrant spermatogenesis (142). Moreover, acetylation is controlled by two types of enzymes viz. acetyltransferases and deacetyltransferases, where acetyltransferases activate gene expression and deacetyl transferases inhibit gene expression (143). Besides, phosphorylation on serine residues leads to gene activation while phosphorylation on the histone variant causes chromatin condensation and gene silencing. On the other hand, during postmeiotic stages of spermatogenesis, RNF8-dependant ubiquitinylation of histones H2A and H2B favors the exchange of histone protein to transition protein (144). Furthermore, deficiency of RNF8 is reported to cause failure in chromatin condensation, loss in sperm motility, reduction in sperm concentration in the epididymis, increased retention of the residual body and reduced capacity to fertilize oocytes (144). In addition, small ubiquitin-like modifiers (SUMOs) also take part in epigenetic modification by regulating gene expression (140).

**Table 2:** Selected molecules involved in post -translational histone modification in relation to fertility.

**Genes**	**Function**	**References**
Suv39h1 (Histone-Lysine N-Methyltransferase, H3 Lysine-9 Specific 1)	Histone methylation (H3)	(128, 129)
LSD1 (Lysine (K) -Specific Demethylase 1)	Histone demethylation (H3)	(130)
HATs (Histone Acetyltransferase 1)	Histone acetylation (H4)	(131)
MYST (MYST lysine acetyltransferases)	Histone acetylation (H4)	(132)
HDACs (Histone Deacetylase 1)	Histone deacetylation (H4)	(131)
SIRT1 (sirtuin family)	Histone deacetylation (H1, H3, H4)	(133)
MUTp	Histone phosphorylation (H2, H3)	(134)
NHK-1	Histone phosphorylation (H2)	(135)
G9a	Histone methylation (H3)	(136)
MSK1, MSK2	Histone phosphorylation (H3)	(137)
PKA	Histone phosphorylation (H3)	(138)
HR6B	Histone ubiquitylation (H2)	(139)
E1 SUMO-activating enzyme 1, E1 SUMO-activating enzyme 2, UBC9	Histone sumoylation (H4)	(140)

### Chromatin Remodeling

Unlike the above two processes of epigenetic modification, chromatin remodeling occurs by shifting of histone proteins along the DNA and consequently results in alteration of sperm physiology. Generally, one phosphate group from an ATP molecule is utilized to loosen the tight packaging of chromatin, thereby allowing DNA-binding factors or other transcription factors to access the unwound DNA and regulate gene expression based on exposure of sequences (145). Furthermore, an additional function of chromatin remodeling is DNA replication and repair of DNA damage. Basically, two ubiquitous families, such as SWI/SNF and the ISWI family are involved in this process.

## Methods for Assessment of Sperm DNA Damage

During the past few decades, a variety of assays has emerged to detect sperm DNA damage and some new tests are under investigation. Briefly, the principle of these techniques is based on incorporation of chromatin probes, mild acid denaturation to the fragmented DNA, staining for the evaluation of sperm DNA damage and oxidation-reduction potential (ORP). Double or single DNA strand breaks are estimated either directly or indirectly. Direct tests comprise the comet assay (single cell gel electrophoresis) forming comet tail and cell head in alkaline or neutral pH conditions, which can be visualized using epifluorescence microscopy after staining. The comet tail length and fluorescence intensity in the cell head determine the degree of DNA fragmentation (146), whereas terminal deoxynucleotidyl transferase dUTP nick-end labeling (TUNEL) assay (147) determines only the fluorescently labeled nucleotides. Apart from these assays, *in situ* nick translation (ISNT) (148) and DNA breakage detection fluorescence *in situ* hybridization (DBD-FISH) (149) and ORP (150) are some of the emerging assays for sperm DNA fragmentation. Indirect tests include DNA denaturability, such as the sperm chromatin structure assay (SCSA) (24, 151), Acridine orange (AO), sperm chromatin dispersion (SCD) test (152), and toluidine blue assay (153). These tests make use of DNA intercalating dyes to emit green to red fluorescence based on the attachment to double/single strand fragmented DNA, whereas staining the lysine residue of histones with CMA3 flurochrome competes for the protamine binding site and thus efficiently detects sperm chromatin integrity and packaging. Since protamine deficiency is likely to be one of the contributing factors to DNA instability and damage, which can affect bull fertility, Fortes et al. (84) assessed protamine deficiency in bull spermatozoa using the sperm protamine deficiency assay (SPDA) and concluded that sperm protamine content and sperm DNA damage are closely associated. Very recently, MiOXSYS System has come in to existence as a novel technology to detect ORP in human semen samples, because ORP has a positive correlation between the proportion of abnormal sperm heads and sperm DNA fragmentation (154, 155). On the other hand, estimation of DNA adducts, 8-OHDG level by liquid chromatography (156) and Raman spectroscopy to fingerprint the chemical composition of semen sample (157), can also be used to predict %DFI. Furthermore, assessment of global DNA methylation level has recently become possible, but the correlation with sperm parameters is yet to be defined (158). The ability of these techniques to estimate sperm DNA damage accurately depends on many technical and biological aspects. The principles, advantages and limitations of a few commonly used tests for sperm DNA damage are summarized in Table 3.

**Table 3:** Different methods for detection of DNA damage in spermatozoa.

**Assay**	**Method**	**Principle**	**Advantages**	**Limitations**	**References**
Sperm chromatin structure assay	Flowcytometry	Measure the DNA denaturation (acid/heat) based on the metachromatic shift from green to red fluorescence	The SCSA accurately estimates the percentage of DNA-damaged sperm	Requires expensive instrumentation (flow cytometer) and HIGHLYorthochromatic staining Skilled technicians	(24)
Sperm chromatin dispersion test	Fluorescence Microscopy	Differentiate between fragmented and non-fragmented DNA based on the presence of peripheral halo dispersion in DNA loops.	Simple, fast, and reproducible, and results are comparable to those of the SCSA. Does not require expensive instrumentation.	Recently introduced test, thus little is known about its limitations and its clinical significance	(159)
Toluidine	Microscopy	Used for metachromatic and orthochromatic staining of chromatin. This stain is a sensitive structural probe for DNA structure and packaging.	Simple and inexpensive and have the advantage of providing permanent preparations for use on an ordinary microscope	Inherent limitation of repeatability dictated by dye equilibrium variations and only limited number of cells can be reasonably scored	(160)
Chromomycin A3	Fluorescent Microscopy/Flow cytometry	Chromomycin A_3_ and protamines compete for the same binding sites in the DNA. High CMA3 fluorescence indicates low protamination state of spermatozoa	The CMA3 assay yields reliable results as it is strongly correlated with other assays used in the evaluation of sperm chromatin	Observer subjectivity may hinder the results if fluorescent microscopy is used. Expensive instrumentation, if flow cytometry is used	(161)
Acridine orange	Fluorescent Microscopy/ flowcytometry	Measures the susceptibility of sperm nuclear DNA to acid-induced denaturation *in situ* by quantifying the metachromatic shift of AO fluorescence from green (native DNA) to red (denatured DNA)	The AO assay is a biologically stable measure of sperm quality. The inter-assay variability is >5%, rendering the technique highly reproducible	Observer subjectivity may hinder the results if fluorescent microscopy is used expensive instrumentation, if flow cytometry is used	(162–164)
Aniline	Microscopy	Discriminates between lysine-rich histone and cysteine/arginine-rich protamine	Simple and inexpensive and have the advantage of providing permanent preparations for use on an ordinary microscope	Inherent limitation of repeatability dictated by dye equilibrium variations and only limited number of cells can be reasonably scored	(160)
TUNEL	Flowcytometry/ Fluorescence Microscopy	The TUNEL assay quantifies the incorporation of dUTP at single- and double-strand DNA breaks	The assay demonstrated fairly good quality control parameters. The intra-observer variability was found to be <8% and the interobserver variability was <7%	Expensive instrumentation, if flow cytometry is used	(165)
Comet	Fluorescent Microscopy	Quantifies the actual DNA damage in both alkaline and neutral method based on tail length.	The comet is a simple and well-standardized low-cost assay that correlates significantly with TUNEL and SCSA assays	The assay requires an experienced observer to analyze the slides and interpret the results	(166, 167)
*In situ* nick translation (INST)	Fluorescent Microscopy	Specifically stains spermatozoa that contain appreciable and variable levels of endogenous DNA damage.	The advantage of the NT assay is that the reaction is based on direct labeling of termini of DNA breaks, and thus the lesions that are measured are identifiable at the molecular level	Expensive instrumentation, if flow cytometry is used	(168)
8-OHDG	Liquid chromatography	It is the most commonly studied biomarker for oxidative DNA damage. Estimates the level of DNA adducts which generate DNA strand breaks.	Specificity and sensitivity are high.	Although 8-OHdG is a potential marker for oxidative DNA damage, artifactual oxidation of dG can occur during the analysis, which can lead to inaccurate results	(156)
Oxidation-reduction potential (ORP)	MiOXSYS System	Direct measure of Oxidative Stress in semen sample, as it describes the relative proportions of oxidants (ROS) to reductants (antioxidants)	Reproducibility of the results are high	Very recently introduced test, thus little is known about its limitations and its clinical significance	(154)

Several studies compared different assays for assessment of sperm DNA fragmentation. However, in spite of intensive research, no consensus has yet been reached as to which tests are most predictive of fertility. In men, the alkaline COMET assay was reported to be the best in predicting male infertility, followed by TUNEL, SCD, and SCSA, whereas the neutral COMET assay had no predictive power (169). Martínez-Pastor et al. (170) compared SCSA and SCD for assessing the chromatin status in bulls and reported that SCD had lower repeatability compared to SCSA. In stallions, Serafini et al. (171) observed that both the neutral comet assay and the SCSA could successfully identify sperm DNA quality. Several studies suggested that the SCSA assay may be an important assay for identification of men (9, 172, 173), bull (14, 94, 174, 175), boar (86, 176), and ram (177) with potentially lowered fertility. Using the SCSA assay, the threshold values for farm animals have been established. The estimated threshold above which the SCSA %DFI has a detrimental impact on fertility varies across species e.g., pigs, 6%; bulls 10–20%; horses: ~28%; men 25–30% (178). In the same context, Kumaresan et al. (13) developed a bull fertility prediction model based on PCA analysis of %DFI and some other seminal attributes, which discriminated above average bulls from below average fertility bulls with a fair degree of accuracy (*R*^2^ = 0.83).

## Prognostic Value of Sperm DNA Quality

With the breakthrough in sperm chromatin quality assessment techniques, the prognostic value of sperm DNA quality has improved, in contrast to routine semen analysis (37, 178). Several studies traced the clinical significance of various DNA fragmentation assays in semen quality and fertility in men (89, 179–181), bull (13, 174, 182), boar (86, 176), and stallion (183). Normally, reductions in fertilization rate, embryo quality, implantation rate, chromosomal aberration, and repeated abortions are associated with sperm DNA fragmentation. Several studies indicated the usefulness of sperm DNA integrity as a tool for fertility assessment (13, 81, 178, 182). In contrast, %DFI was reported to have a low impact on fertilization (184), implantation rates (185), and embryo quality (186). In the case of bulls, Castro et al. (83) reported that protamine deficiency in bovine spermatozoa may not have a strong biological significance to explain the fertility difference among individual bulls. Therefore, as of now, the prognostic value of DNA fragmentation remains controversial, as it is coupled with many other factors, such as semen collection and processing procedures, insemination technique and method of assessment of DNA fragmentation (TUNEL, SCSA, SCD, AO, and COMET assay). Besides, several researchers showed seminal parameters to be within normal ranges (such as motility, concentration and morphology) with varying degrees of sperm DNA fragmentation assessed using SCSA &TUNEL assays, but reported compromised fertilization (80, 151, 187). Values of %DFI above 30% in infertile men, 28% in the stallion and 10–20% in bulls, were reported to be associated with a failure to maintain pregnancy although the sperm were able to fertilize oocytes. Karoui et al. (174) also related %DFI values of 7 to 10% with a poor success rate of Artificial Insemination (AI) in bulls. Although several studies on sperm DFI in men are available (9, 11, 89), discriminating between fertile and sub-fertile individuals, few reports are available on farm animals. In a study evaluating sperm DNA damage in bulls and men of known fertility, it was found that infertile bull sperm showed 1.6-fold higher DNA fragmentation rates compared to bulls of proven fertility, and sperm from infertile men showed a 2.25-fold increase in sperm DNA fragmentation compared to fertile men (24). In a recent study, %DFI was negatively and significantly correlated to bull fertility; spermatozoa from below average fertility bulls showed 2.16-fold higher %DFI as compared to above-average fertility bulls (12). After analyzing the trends in sperm DNA fragmentation assessment over a period of 20 years, Baskaran et al. (188) concluded that sperm DNA fragmentation was predominantly investigated in relation to lifestyle and a few infertile conditions, and a substantial increase in research is warranted to establish sperm DNA fragmentation as a prognostic/diagnostic parameter to evaluate clinical scenarios and ART outcomes in human beings.

## Bottom Line

Traditional routine semen analysis has limited value for the prediction of sperm fertilizing potential. The prognostic value of the currently available *in vitro* tests for male fertility prediction is also limited. The data accumulated over the last four decades, mostly on men, indicate that DNA integrity correlates well with sperm fertility potential. Furthermore, the majority of the studies indicates that DNA damage assessment has a better predictive score than conventional semen analysis. In the era of assisted reproductive techniques (ART), evaluation of the quality of the sperm genome and selection of good quality spermatozoa assume much significance for desirable pregnancy outcome. The assessment of sperm DNA damage in ART, such as *in vitro* embryo production, is important because these techniques bypass the natural selection barriers of the female reproductive tract. When spermatozoa with a lot of DNA damage are used, the possibilities of transmitting genetic aberrations to the embryo/fetus are high. It has been demonstrated that assessment of the sperm DNA fragmentation index and selection of spermatozoa with low %DFI prior to ART, improved fertilization rates. Several studies indicate that sperm DNA damage is negatively associated with male fertility, fertilization, and embryonic development. A recent report indicated that sperm %DFI was significantly higher (*P* < 0.01) in male factor infertility compared to either female factor or unknown factor infertility (12). Therefore, the inclusion of the DNA fragmentation index in breeding soundness evaluation could be a better prognostic value for selection of quality breeding males for an artificial breeding programme.

On the research front, we still have a long way to go to understand fully the impact of fertilization of an oocyte by a spermatozoon with damaged DNA. The majority of the earlier studies reported the relationship of sperm DNA damage with fertility but limited information is available on the molecular details of how damaged sperm DNA affects fertilization and post-fertilization embryo/fetus development. In addition, the long-term consequences of using sperm with compromised DNA integrity are unknown. To make it possible to assess sperm DNA in frozen semen stations, a precise and simplified novel method is required. Whatever the assay to be developed or refined, it should allow assessment of sperm DNA integrity without destruction of the fertilizing potential of spermatozoa so that the same germ cell could be used for assessment and subsequently for fertilization; this would provide a safe and effective diagnostic method in cases opting for ART.

## Conclusion

There is consensus among the researchers that DNA damage is greater in infertile males compared to fertile males in several species. The available literature also convincingly shows that sperm DNA damage influences the fertility outcome to a variable degree. Although several methods are used to assess sperm DNA damage, the different types of DNA defects and inconsistency in reproducibility of results, indicate that it may be necessary to use a combination of tests for reliable and reproducible results. However, it is noteworthy that the majority of the information generated was from men; large-scale studies using appropriate samples/methodologies are very limited in farm animals. Although, a substantial increase in research is warranted to establish sperm DNA damage as a prognostic/diagnostic parameter to evaluate male fertility in farm animals, based on the information available, across the species, it may be inferred that incorporation of sperm DNA fragmentation assay in the breeding soundness evaluation could improve the accuracy of selection of quality breeding males for an artificial breeding programme.

## Author Contributions

AK and JM conceptualized the review. All of the authors were involved in literature review, development of the manuscript, and approved the manuscript for publication.

## Conflict of Interest

The authors declare that the research was conducted in the absence of any commercial or financial relationships that could be construed as a potential conflict of interest.
